# Diagnosis, clinical features and survival analysis of invasive pulmonary aspergillosis among critically ill patients: a retrospective cohort study based on updated criteria

**DOI:** 10.3389/fcimb.2026.1732182

**Published:** 2026-02-23

**Authors:** Liufang Gao, Shuiwen Li, Xiaolong Huang, Weizhe Huang, Zhu Chen, Yaogui Ning

**Affiliations:** 1 Department of Intensive Care Unit, The First Affiliated Hospital of Xiamen University, School of Medicine, Xiamen University, Xiamen, China; 2 The Graduate School of Fujian Medical University, Fuzhou, Fujian, China

**Keywords:** diagnosis, EORTC/MSG criteria, FUNDICU consensus, intensive care unit (ICU), invasive pulmonary aspergillosis (IPA), mortality, risk factors, treatment

## Abstract

**Objective:**

To evaluate the incidence, diagnostic performance, clinical characteristics, and prognostic determinants of invasive pulmonary aspergillosis (IPA) in critically ill patients, using updated EORTC/MSG and FUNDICU criteria.

**Methods:**

We retrospectively analyzed a heterogenous ICU pneumonia cohort between Jan 2022 and Jun 2025. All included patients underwent at least one mycological assessment. IPA was diagnosed based on integrated EORTC/MSG and FUNDICU criteria. Diagnostic accuracy of mycological assays was evaluated, and clinical profiles were compared between IPA and non-IPA patients. Independent risk factors for mortality were identified via multivariate Cox regression.

**Results:**

Among 1835 patients, the IPA incidence was 7.1% (n=131). Of all mycological assays, bronchoalveolar lavage fluid galactomannan (BALF GM) demonstrated the highest sensitivity (85.5%) with robust specificity (82.9%). Molecular testing combined with BALF GM yielded a sensitivity of 64.4% and a specificity of 80.0%. Serum GM exhibited the lowest sensitivity (41.7%) but the highest specificity (89.2%); its combination with culture or molecular methods further enhanced specificity to >95%. Although patchy infiltrates were the most frequent radiological findings in both groups, consolidation (63.4% vs. 40.3%, *P* < 0.001), nodules (22.9% vs. 9.1%, *P* = 0.014), cavities (17.6% vs 3.9%, *P* = 0.004), and tree-in-bud signs (9.9% vs. 1.3%*, P* = 0.019) were significantly more prevalent in IPA patients. IPA patients experienced longer ICU stays (median 20 vs. 16 days) and significantly higher ICU mortality (55.0% vs 26.0%). Multivariable analysis identified IPA (HR: 2.064, 95% CI = 1.211-3.518, *P* = 0.008) and a prolonged interval from admission to the first positive mycological test (HR: 1.017, 95% CI = 1.004-1.031, *P* = 0.011) as independent risk factors for death. Conversely, anti-*Aspergillus* therapy (HR: 0.489, 95% CI = 0.275-0.871, *P* = 0.015) and tracheostomy (HR: 0.351, 95% CI = 0.222-0.557, *P* < 0.001) were associated with improved survival.

**Conclusion:**

IPA is a common and lethal complication in ICU pneumonia patients, serving as an independent predictor of mortality. BALF GM, particularly when integrated with molecular testing, is the most effective mycological diagnostic method. Reducing the diagnostic window, initiating timely antifungal therapy, and proactive airway management (e.g., tracheostomy) may significantly improve clinical outcome for these critically ill patients.

## 1 Introduction

Invasive aspergillosis (IA) is a significant global health concern with an annual incidence exceeding two million cases. According to the latest literature, which does not include data from the influenza or COVID-19 outbreaks, it predominantly affects individuals with chronic obstructive pulmonary disease (COPD) and critically ill patients admitted to the intensive care unit (ICU) (Denning, 2024). IA already demonstrates the highest incidence and crude mortality rate among all invasive fungal diseases. Without effective treatment, the case fatality rate can exceed 95%. Compounding this severity, the absence of broad diagnostic criteria and low clinical awareness result in a substantially lower treatment rate among COPD and ICU patients compared to hematological populations (Denning, 2024).

Invasive pulmonary aspergillosis (IPA), the most common form of IA, was once thought to be an opportunistic infection in immunocompromised patients with leukemia or neutropenia (Donnelly et al., 2020). Nowadays, IPA is increasingly reported in ICU patients without classical predisposing immunodeficiency, including patients with COPD, severe liver disease, and severe viral pneumonia caused by COVID 19 or influenza (Schauwvlieghe et al., 2018; Guo et al., 2023; Vanderbeke et al., 2023; Feys et al., 2024; Epelbaum et al., 2025).

However, it is notably complex and challenging to diagnose IPA in ICU patients because of high heterogeneity among them, as well as concerns about the feasibility, safety and accuracy of necessary work-up (Scharmann et al., 2023; Garnacho-Montero et al., 2024; Hu et al., 2024). Moreover, ICU patients with IPA often present with atypical clinical manifestations and radiologic finding because of the presence of multiple comorbidities and the frequent absence of neutropenia (Gaffney et al., 2023; Ledoux and Herbrecht, 2023). Due to recent advances in the diagnosis and management of IPA, several criteria have been developed and updated in recent decades to address the limitations of the consensus definitions proposed prior to 2021 by the European Organization for Research and Treatment of Cancer and Mycosis Study Group Education and Research Consortium (EORTC/MSGER) (Gaffney et al., 2023; Ledoux and Herbrecht, 2023). For better generalizability, the Invasive Fungal Diseases in Adult Patients in ICU (FUNDICU) project published a standard set of definitions for IPA in non-neutropenic, ICU patients lacking classical immunocompromised host factors, as a complement rather a replacement for the previous suggested criteria (Bassetti et al., 2024).

Nevertheless, several studies have shown that there is a lack of consistency between the various diagnostic criteria (Schroeder et al., 2022; Liu et al., 2023). How the recently revised criteria will impact IPA diagnosis and treatment in ICU patients remains unknown. The aim of the study was to explore the clinical practice of IPA diagnosis and treatment for ICU patients in real world, as well as the effects of revised criteria.

## 2 Methods

### 2.1 Study participants and design

**Figure 1:**
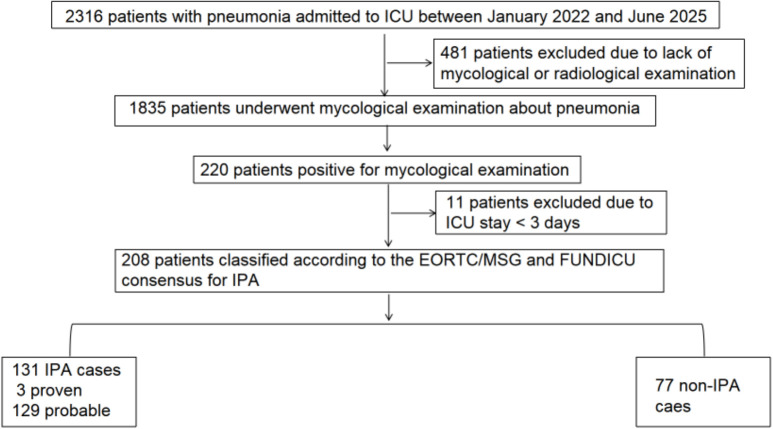
Flowchart of patient included in the study. ICU, intensive care unit; FUNDICU, Invasive Fungal Diseases in Adult Patients in ICU; IPA, invasive pulmonary aspergillosis; EORTC/MSG, European Organization for Research and Treatment of Cancer Mycoses Study Group Education and Research Consortium.

This retrospective study was conducted at the General Intensive care unit (ICU) of the First Affiliated Hospital of Xiamen University. We screened a total of 2,316 patients with suspected pneumonia admitted between January 2022 and June 2025. To be eligible for inclusion, patients had to meet the following criteria: (1)new onset or worsening cough or dyspnea with or without fever and expectoration; the presence of new or progressive radiographic pulmonary infiltrates; (2) age ≥ 18 years; (3) an ICU length of stay ≥ 3 days; (4) mycological evidence of *Aspergillus*, defined by at least one positive result from fungal culture, galactomannan (GM) assay, polymerase chain reaction (PCR); and (5) availability of chest computed tomography (CT) imaging data. Patients who did not meet these criteria were excluded. Ultimately, 208 patients were enrolled in the final analysis (**Figure 1**).

### 2.2 Data collection

We extracted the following variables from electronic medical records: patient demographics, comorbidities, host factors, medication use, mycological results, and the initial Sequential Organ Failure Assessment (SOFA) score. Additionally, we documented information on bronchoscopy, tracheostomy, mechanical ventilation, extracorporeal membrane oxygenation (ECMO), antifungal treatment, and the cumulative prednisone equivalent during hospital stay. Clinical outcomes included ICU and hospital length of stay as well as survival status.

Chest CT images were assessed by a senior ICU physician and a radiologist, with any disagreements resolved by consensus or by a third senior radiologist.

### 2.3 Diagnostic criteria

ICU patients are substantially heterogeneous. Therefore, we employed both the EORTC/MSG definition and the FUNDICU consensus (Donnelly et al., 2020; Bassetti et al., 2021, Bassetti et al., 2024) to diagnose probable IPA in critically ill patients with a combination of host factors, clinical criteria, and evidence of *Aspergillus*.

1. Host factors included both classic immunocompromised conditions defined by EORTC/MSG consensus and several risk factors expanded for ICU patients in recent years, as follows:

- Glucocorticoid treatment with prednisone equivalent of 20 mg or more per day, or equivalent cumulative doses.
- Qualitative or quantitative neutrophil defects (inherited neutrophil deficiency, absolute neutrophil count of ≤500 cells/mm^3^).
- Chronic respiratory airway abnormality (chronic obstructive lung disease, bronchiectasis).
- Decompensated cirrhosis.
- Severe viral pneumonia caused by influenza virus or COVID-19.

2. Clinical criteria were based on the presence of at least one of the following:

- Imaging signs presented in EORTC/MSG criteria: dense, well-circumscribed lesions with or without a halo sign; air-crescent sign; cavity not attributable to other causes.
- Any other infiltrate on pulmonary imaging.
- Bronchoscopy findings: tracheobronchial ulceration, nodule, pseudomembrane, plaque, or eschar.

3. *Aspergillus* evidence was established by at least one of the following tests:

- A positive Aspergillus culture from the lower respiratory tract.
- A GM optical index on bronchoalveolar lavage fluid (BALF) of ≥1 on serum sample of ≥0.5 using the Platelia Aspergillus enzyme immunoassay (Bio-Rad Laboratories, Munich, Germany) according to the manufacturer’s instructions.
- Two or more duplicate PCR tests positive for *Aspergillus* spp. on BALF samples, which was defined as a cycle threshold (Ct) value <40 in accordance with the manufacture’s protocol.

Patients were classified as either IPA or non-IPA based on whether they met the above diagnostic criteria. Only three patients were pathologically confirmed as proven IPA.

### 2.4 Statistical analysis

Due to the non-normal distribution, continuous variables are presented as median with interquartile range and were compared by Mann–Whitney U test. Categorical data are expressed as numbers, percentages and compared using the chi-square test. To identify independent risk factors for survival, variables with a P value < 0.1 in univariable COX regression were included in a multivariable Cox regression model. A two-sided P value < 0.05 was deemed statistically significant in the final model. Regarding missing data, complete case analysis was applied for the statistical analysis of individual variables (i.e., cases with missing values were excluded), and all variables included in the multivariable Cox regression analysis had no missing values. All statistical analyses were performed using the Statistical Package for the Social Sciences statistical software (version 26.0; SPSS Inc, Chicago, IL, USA). Figures were designed using Prism version 10.6.

## 3 Results

### 3.1 Study population

After excluding patients without mycological workup or those with an ICU stay of less than 3 days, *Aspergillus* spp. was identified in 208 of the 1,835 screened patients. Among these 208 cases, the etiologies of pneumonia were bacterial (56.3%), mixed (21.6%), viral (4.3%) and unknown (17.8%). All patients with pneumonia in both groups received standard antibiotic therapy, and no statistically significant difference was observed in the antibiotic use profile between the two groups. Based on the updated EORTC/MSG and FUNDICU consensus, 131 cases were diagnosed as IPA, representing an incidence of 7.1% among the enrolled cohort.

As shown in **Table 1**, the IPA group demonstrated significantly higher rates of solid tumors (34.4% vs. 16.9%, *P* = 0.007), immunosuppressive therapy (75.6% vs. 35.1%, *P* < 0.001), and neutropenia (12.2% vs. 1.3%, *P* = 0.004) than the non-IPA group. Furthermore, IPA patients received higher cumulative corticosteroid doses (prednisone equivalents) during their hospital stay. Conversely, demographics, comorbidities, and admission SOFA scores were similar between groups. Mycologically, significantly more IPA patients tested positive in ≥ 2 different assays (67.9% vs. 16.9%, P < 0.001).

**Table 1:** Clinical characteristics and managements of patients and comparison between two groups.

Variables	All (n=208)	non-IPA (n=77)	IPA (n=131)	*P* value
Age, median (IQR)	71 (59,79)	71(55.0,78.0)	70 (63,80)	0.664
Male, n (%)	150 (72.1)	58 (75.3)	92 (70.2)	0.522
Comorbidities				
Diabetes, n (%)	62 (29.8)	22 (28.6)	40 (30.5)	0.875
CKD, n (%)	25 (12.0)	6 (7.8)	19 (14.5)	0.188
Autoimmune disease, n (%)	33 (15.9)	7 (9.1)	26 (19.8)	0.049
Chronic pulmonary disease^*^, n (%)	53 (25.5)	14 (18.2)	39 (29.8)	0.071
Decompensated cirrhosis, n (%)	7 (3.4)	1 (1.3)	6 (4.6)	0.263
EORTC/MSG host factors				
Solid tumor, n (%)	58 (27.9)	13 (16.9)	45 (34.4)	0.007
Hematologic malignancy, n (%)	9 (4.3)	1 (1.3)	8 (6.1)	0.158
Immunosuppressive treatment^#^, n (%)	126 (60.6)	27 (35.1)	99 (75.6)	<0.001
Glucocorticoid^&^ (mg, IQR)	445.8 (150.0, 1050.0)	158.3 (33.3, 492.5)	600 (266.7, 1366.7)	<0.001
Neutropenia, n (%)	17 (8.2)	1 (1.3)	16 (12.2)	0.004
SOFA score, median (IQR)	8.0 (5.0, 11.0)	8.0 (5.0, 10.0)	9.0 (6.0, 12.0)	0.169
Bacterial pneumonia, n (%)	162 (77.9)	54 (70.1)	108 (82.4)	0.039
Viral pneumonia, n (%)	55 (26.4)	0 (0.0)	55 (42.0)	<0.001
Mycological findings				
Positive culture, n (%)	127/207 (61.4)	56/76 (73.7)	71/131 (54.2)	0.007
Positive BALF GM, n (%)	107/158 (67.7)	7/41 (17.1)	100/117 (85.5)	<0.001
Positive serum GM, n (%)	60/191 (31.4)	7/65 (10.8)	53/126 (42.1)	<0.001
Positive molecular test, n (%)	68/102 (66.7)	22/35 (61.1)	46/66 (69.7)	0.389
≥ 2 kinds of positive test, n (%)	101 (48.6)	12 (15.6)	89 (67.9)	<0.001
Radiological findings, n (%)				
Consolidation	114 (54.8)	31 (40.3)	83 (63.4)	0.001
Nodule	37 (17.8)	7 (9.1)	30 (22.9)	0.012
Cavity	26 (12.5)	3 (3.9)	23 (17.6)	0.004
Tree-in-bud sign	14 (6.7)	1 (1.3)	13 (9.9)	0.019
Patchy infiltrates	158 (76.0)	54 (70.1)	104 (79.4)	0.131
Bronchial wall thickening	53 (25.5)	15 (19.5)	38 (29.0)	0.128
Pleural effusions	76 (36.5)	30 (39.0)	46 (35.1)	0.578
ICU management				
IMV, n (%)	189 (90.9)	67 (87.0)	122 (93.1)	0.211
CRRT, n (%)	106 (51.0)	31 (40.3)	75 (57.3)	0.022
vv-ECMO, n (%)	13 (6.3)	1 (1.3)	12 (9.2)	0.034
Tracheostomy, n (%)	100 (48.1)	33 (42.9)	67 (51.1)	0.255
Anti-*aspergillus* treatment, n (%)	165 (79.3)	47 (61.0)	118 (90.1)	<0.001

Continuous variables are given as median (1st to 3rd quartile), categorical variables are given as n (%).

*Chronic pulmonary disease includes chronic obstructive pulmonary disease, bronchiectasis, pulmonary fibrosis, etc.

^#^immunosuppressive treatment includes high-dose corticosteroids (at a mean minimum dose of 0.3 mg/kg/day of prednisone equivalent for > 3 weeks, or equivalent cumulative doses) or other immunosuppressants (during the past 3 months); chemotherapy during the past 6 months or chest radiotherapy during the past 1 month.

^&^Glucocorticoid: the cumulative prednisone equivalent during hospital stay.

CKD, chronic kidney disease; SOFA, Sequential Organ Failure Assessment Score; BALF, bronchoalveolar lavage fluid; GM, galactomannan; ICU, intensive care unit; IMV, invasive mechanical ventilation; CRRT, continuous renal replacement therapy; vv-ECMO, veno-venous extracorporeal membrane oxygenation.

In terms of ICU management, IPA patients more likely to receive CRRT (57.3% vs. 40.3%, *P* = 0.022), vv-ECMO (9.2% vs 1.3%, *P* = 0.034), and anti-*aspergillus* treatment (90.1% vs. 61.0%, *P* < 0.001).

### 3.2 Mycological assays

**Figure 2:**
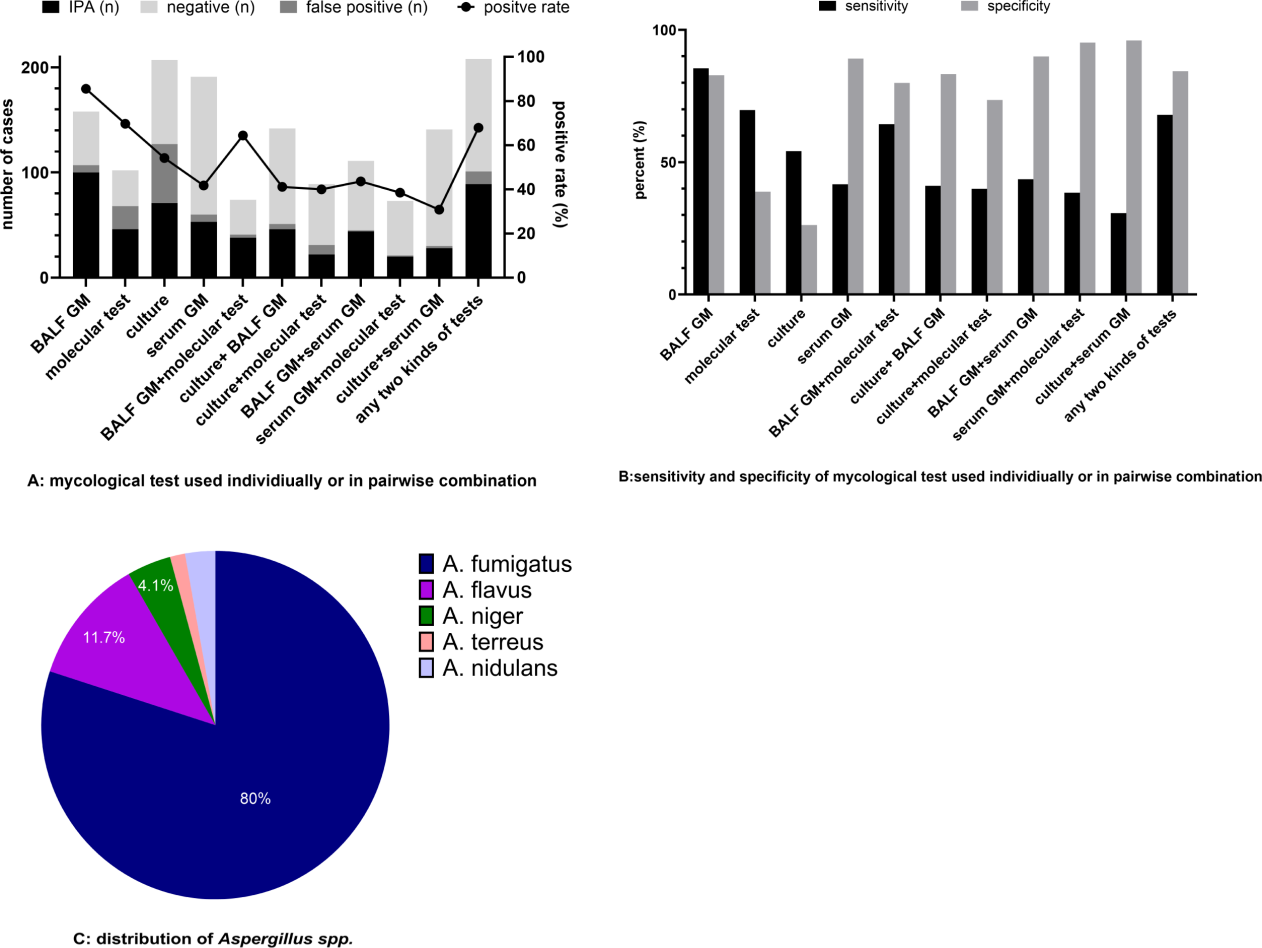
The results of different mycological tests and *Aspergillus* categories. **(A)** The results of different mycological tests, with black bars representing the number of IPA cases diagnosed according to EORTC/MSG criteria plus FUNDICU consensus. **(B)** The sensitivity and specificity of different methods. **(C)** Distribution of *Aspergillus* spp. IPA rate: The proportion of clinically diagnosed IPA patients (mostly probable) among each type of mycologically positive cases. BALF, Bronchoalveolar lavage fluid; GM, Galactomannan.

**Figure 2** provides a summary of the performance of different mycological assays, either used separately or in combination. Culture from LRTs was the most frequently employed method (207/208), followed by serum GM (191/208) and BALF GM (158/208). BALF GM showed the highest positive rate (107/158, 67.7%), followed by molecular tests (68/102, 66.7%). On the other hand, serum GM demonstrated the lowest positive rate (60/191, 31.4%).

The combination of any two methods produced an overall positive rate of 48.6%, with BALF GM plus molecular testing yielded the highest positive rates (55.4%) as shown in **Figure 2A**.

**Figure 2B** shows the sensitivity and specificity of different mycological methods, used individually or in pairwise combinations, for the diagnosis of IPA. BALF GM exhibited the highest sensitivity (85.5%) with the specificity of 82.9%. Low sensitivity and specificity (54.2% and 26.3% respectively) were obtained from culture. The combination of BALF GM and molecular test showed a moderate level of sensitivity (64.4%) and specificity (80.0%). Although serum GM showed limited sensitivity (41.7%), its specificity was considerably high (89.2%). Furthermore, specificity rose to more than 95% when serum GM was used in combination with either culture or molecular methods.

A. fumigatus accounted for 80% of the distribution of *Aspergillus* species, followed by *A.* flavus (11.7%), as shown in **Figure 2C**.

### 3.3 CT imaging manifestations

**Figure 3:**
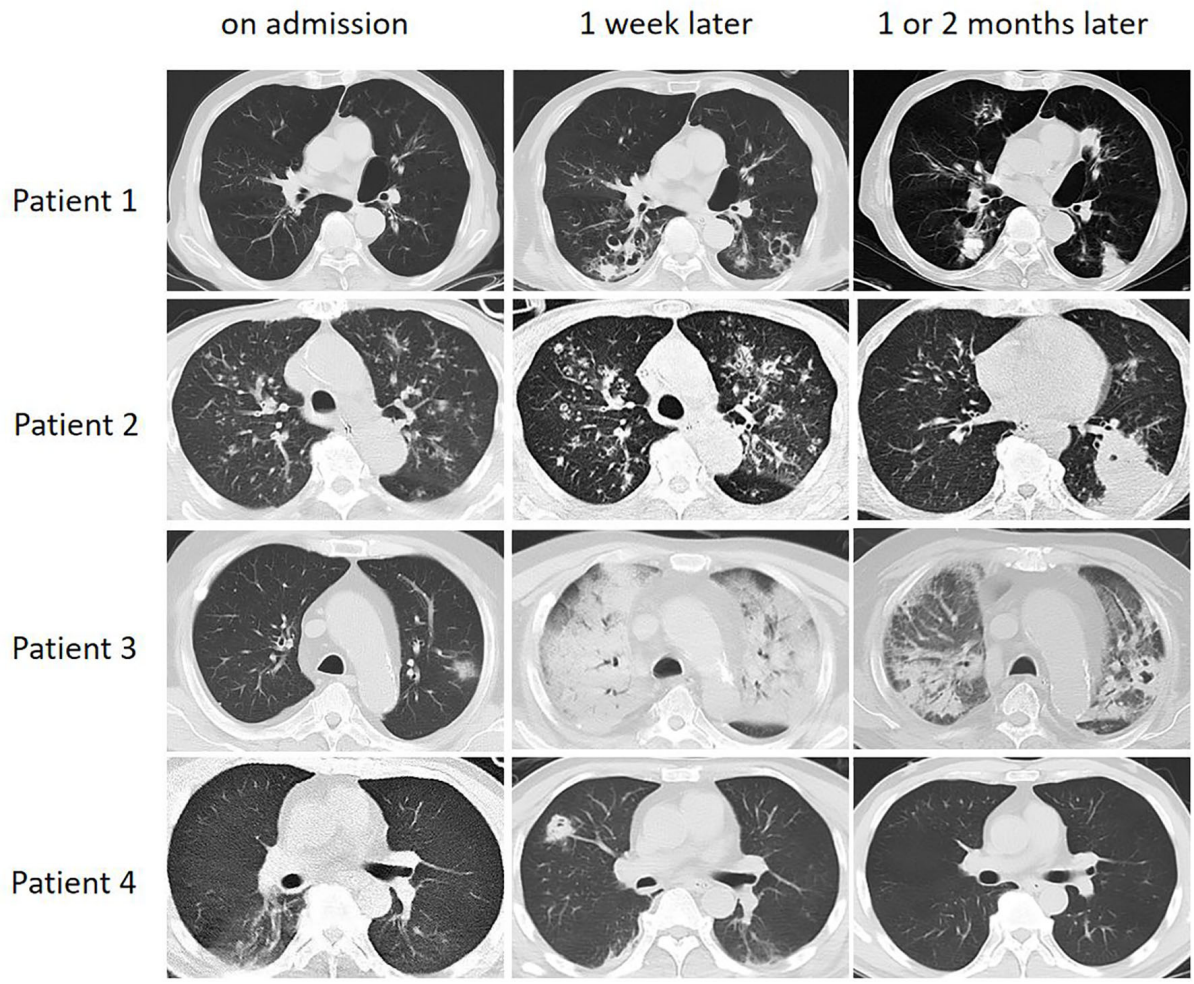
Serial CT imaging of four representative non-neutropenic IPA cases. • Patient 1: A 62-year-old male with COPD. The initial CT showed bronchial wall thickening, followed 1 month later by the development of multiple thin-walled cavities and wedge-shaped consolidation. • Patient 2: A 70-year-old male with COPD and influenza infection. The initial CT revealed a tree-in-bud pattern. One month later, a new consolidation with a small cavity appeared in the dorsal side of the left lung lobe. • Patient 3: A 60-year-old male with diabetes and severe viral pneumonia. CT at 1-week post-admission demonstrated diffuse bilateral consolidation, which subsequently evolved into multiple thick-walled cavities. • Patient 4: A 53-year-old male with diabetes as the sole risk factor. CT performed 1 week after admission showed isolated nodules, which had resolved completely on follow-up imaging 2 months later.

Radiological characteristics of the study population are summarized in **Table 1**. Patchy infiltrates were the most frequent manifestation; however, no significant difference was observed between the IPA and non-IPA groups (79.4% vs. 70.1%, *P* = 0.135). In contrast, the prevalence of consolidation (63.4% vs. 40.3%, *P* < 0.001), and nodules (22.9% vs. 9.1%, *P* = 0.014) was significantly higher in the IPA group. Although less common, cavities (17.6% vs. 3.9%) and tree-in-bud signs (9.9% vs. 1.3%) also showed statistically significant intergroup differences (all *P* < 0.05). No significant disparities were identified between the two groups regarding bronchial wall thickening or pleural effusions. **Figure 3** illustrates the chest CT manifestations of four representative patients with IPA at different disease stages.

### 3.4 Outcome and survival analysis between two groups

**Figure 4:**
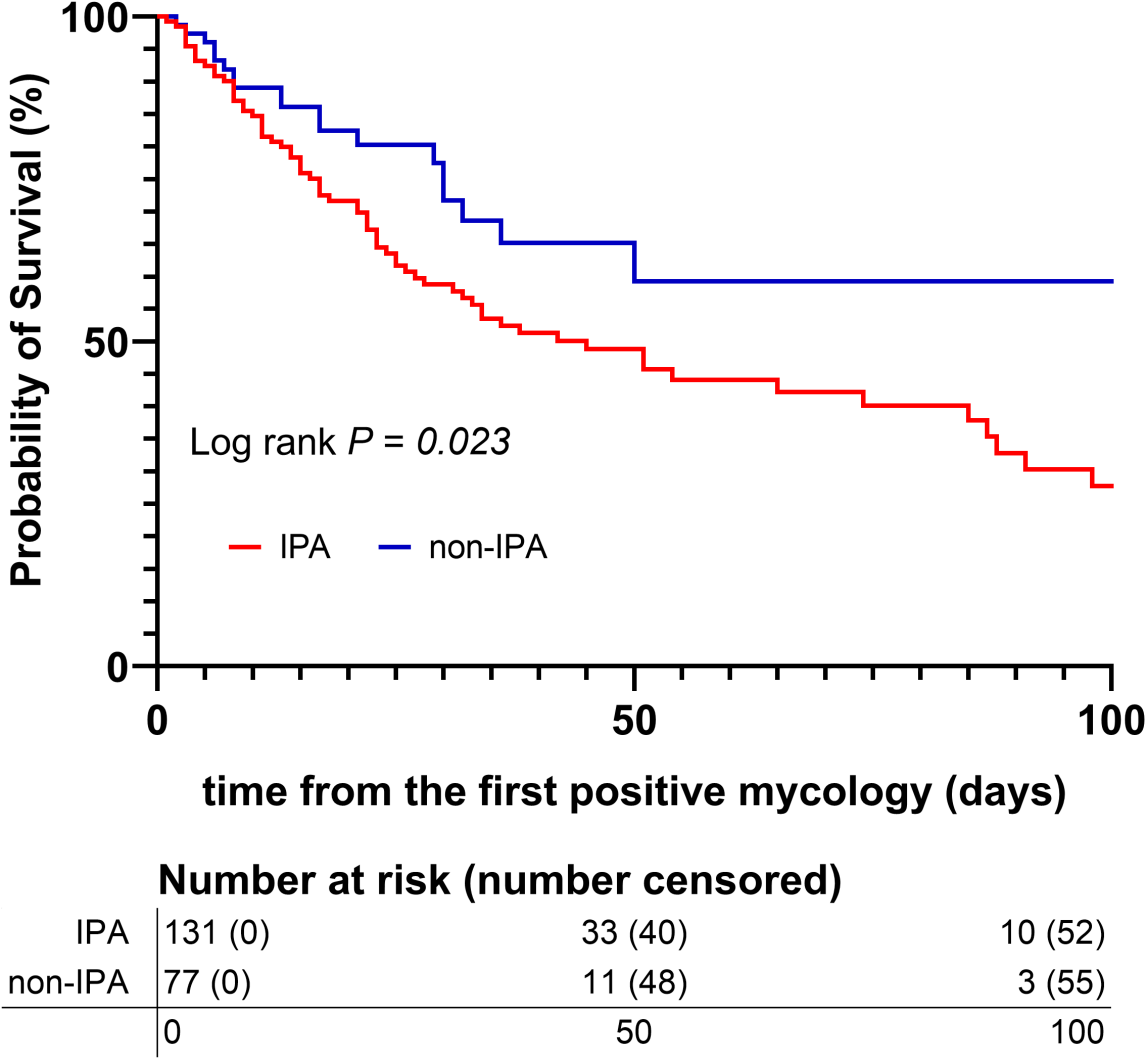
Kaplan - Meier survival curve.

**Table 2** summarizes that clinical outcomes were significantly worse in the IPA group. These patients had a longer median ICU stay (20 vs. 16 days, *P* = 0.041) and a significantly longer duration of invasive mechanical ventilation (14.7 vs. 6.9 days, *P* < 0.001). Furthermore, the mortality rate was significantly elevated in the IPA group (55.0% vs. 26.0%, *P* < 0.001). Kaplan-Meier analysis further validated the difference in survival (**Figure 4**). Cox regression analysis revealed that IPA (HR: 2.064, 95% CI = 1.211-3.518, *P* = 0.008) and a longer interval from admission to the first positive mycological test (HR: 1.017, 95% CI = 1.004-1.031, *P* = 0.011) were independent risk factors for survival. However, multivariable analysis showed that tracheostomy (HR: 0.351, 95% CI = 0.222-0.557, *P* < 0.001) and anti-*aspergillus* therapy (HR: 0.489, 95% CI = 0.275-0.871, *P* = 0.015) were associated with significantly reduced mortality (**Table 3**).

**Table 2:** Clinical outcomes of patients and comparison between two groups.

Outcomes	All (n=208)	non-IPA (n=77)	IPA (n=131)	*P* value
ICU stay, median (IQR)	19 (10.0, 33.0)	16 (8.0,27.5)	20 (11.0,36.0)	0.041
IMV days, median (IQR)	11.6 (4.4, 22.8)	6.9 (2.59, 19.65)	14.7 (6.0, 27.7)	<0.001
Hospital days, median (IQR)	33 (21.0, 60.3)	30 (17.5, 47.0)	34 (22.0,65.0)	0.123
ICU mortality, n (%)	92 (44.2)	20 (26.0)	72 (55.0)	<0.001

**Table 3:** Cox regression analysis of risk factors for ICU mortality.

Variable	Cox univariate analysis	P value	Cox multivariate analysis	P value
	HR (95% CI)		HR (95% CI)	
SOFA	1.078 (1.023-1.137)	0.005	1.033 (0.975-1.094)	0.275
Group (IPA vs non-IPA)	1.758 (1.069-2.891)	0.026	2.064 (1.211- 3.518)	0.008
Time from admission to positive mycology	1.015 (1.002-1.028)	0.027	1.017 (1.004-1.031)	0.011
Anti-aspergillus treatment	0.620 (0.359-1.071)	0.087	0.489 (0.275-0.871)	0.015
Requirement for CRRT	2.151 (1.355-3.414)	0.001	1.595 (0.964-2.638)	0.069
Requirement for IMV	6.616 (0.919-47.626)	0.061	5.930 (0.792-44.414)	0.083
Requirement for vv-ECMO	2.196 (1.164-4.143)	0.015	2.004 (1.028-3.905)	0.041
Tracheostomy	0.517 (0.338-0.790)	0.002	0.351 (0.222-0.557)	<.001

## 4 Discussion

This study provides a comprehensive retrospective analysis of IPA in critically ill pneumonia patient using the combined FUNDICU and EORTC/MSG criteria.

### 4.1 Epidemiology and microbiological profile

The observed IPA incidence of 7.1% aligns with the reported 5%–10% in recent literature (Loughlin et al., 2020; Bassetti et al., 2024), though slightly exceeding the 6.5% rate found using 2021 EORTC/MSG ICU criteria alone (Liu et al., 2023). Notably, only 12.2% of our cohort presented with traditional neutropenia, while 75.6% received immunosuppressive therapy, underscoring the shift toward iatrogenic host factors and chronic organ failure recognized by the FUNDICU consensus (Bassetti et al., 2024). *Aspergillus fumigatus* remained the dominant pathogen (80%), consistent with regional findings in China (Feng et al., 2025). The 55% crude mortality rate reinforces IPA as a devastating complication in the ICU (Denning, 2024).

### 4.2 Comparative diagnostic performance of mycological assays

The diagnostic landscape of IPA in the ICU has shifted toward non-culture-based assays due to the limitations of traditional methods. Our findings underscore the superior sensitivity of BALF GM (85.5%), which outperformed both LRT culture (54.2%) and serum GM (41.7%), consistent with previous reports (Scharmann et al., 2023; Hu et al., 2024). As a polysaccharide released during active growth, BALF GM is sampled directly from the infection site, making it the preferred diagnostic tool when bronchoscopy is feasible (Bassetti et al., 2024; Heylen et al., 2024). However, the observed specificity (82.9%) was slightly lower than literature values, likely reflecting the high prevalence of false-positive triggers and population heterogeneity inherent in critically ill cohorts (Garnacho-Montero et al., 2024).

Molecular Testing (PCR) demonstrated the second-highest sensitivity (69.7%), providing particularly valuable for culture-negative cases or patients on empirical antifungal therapy (Hu et al., 2024), despite its limited accessibility in resource-constrained settings. In contrast, serum GM exhibited low sensitivity (41.7) but high specificity (89.2%). This low sensitivity is characteristic of non-neutropenic patients, where fungal antigens often remain localized within the pulmonary parenchyma rather than circulating systemically (Leeflang et al., 2015). Nevertheless, consecutive positive serum GM results remain a robust indicator of IPA, especially when BALF collection is contraindicated (Huang et al., 2021).

**LRT Culture** performed poorly (sensitivity 54.2%, specificity 26.3%), as slow growth. and colonization issues hinder its reliability (Garnacho-Montero et al., 2024; Hu et al., 2024). Consequently, our data support the FUNDICU recommendation for multimodal strategies (Bassetti et al., 2024); specifically, combining BALF GM and PCR yield the most balanced profile (64.4% sensitivity, 80.0% specificity). In scenarios where BALF is unavailable, pairing serum GM with either culture or PCR can enhance specificity. Crucially, our findings caution against over-reliance on LRT culture alone or a single positive PCR result, advocating instead for the integration of multiple mycological markers to guide clinical decision making (Bassetti et al., 2021, Bassetti et al., 2024).

### 4.3 Radiological evolution and clinical context

The radiographic presentation of IPA in our cohort was largely nonspecific, with patchy infiltrates being the most common finding, while characteristic CT signs such as cavities, nodules, and halo sign were infrequent. This variability underscores that CT manifestations of IPA are dynamic and highly heterogeneous, shaped by underlying comorbidities, host immune status, and potential co-infections (Alexander et al., 2021; Brandi et al., 2022).

Our findings suggest a correlation between the extent of CT abnormalities and the severity of immunosuppression. Patients with profound immune impairment (e.g., diabetes complicated by severe viral pneumonia) showed diffuse consolidation, while those with milder factors presented with more localized lesions (Fischer et al., 2022; Lu et al., 2024). Notably, classic cavities often emerged only after one week, suggesting early reliance on “typical” signs may cause diagnostic delays. Furthermore, early airway-invasive signs like the tree-in-bud pattern, often seen in COPD and influenza patients, are critical but frequently overlooked markeres of *Aspergillus* bronchitis (Liu et al., 2020; Alexander et al., 2021; Feys et al., 2024). The resolution of lesions following timely antifungal therapy versus irreversible injury in delayed cases underscores the need for serial CT scans performed at appropriate intervals (Slavin et al., 2021).

### 4.4 Outcomes and modifiable factors

IPA patients faced significantly worse outcomes, including longer ICU stays and nearly double the hospital mortality rate (55.0% vs. 26.0%), consistent with meta-analyses (Loughlin et al., 2020; Denning, 2024). Multivariable Cox regression identified, IPA (HR: 2.064) and delayed diagnosis as independent risks. Conversely, anti-*Aspergillus* therapy (HR: 0.489) was significantly associated with reduced mortality, This supports guideline-based pre-emptive therapy for high-risk, test-positive patients (Ledoux and Herbrecht, 2023; Bassetti et al., 2024) and highlights the consequences of undertreatment, a well-documented problem in COPD and ICU patients due to low awareness (Denning, 2024).

Notably, tracheostomy (HR: 0.351) was associated with significantly lower mortality. This may be related to that patients with IPA often require prolonged mechanical ventilation and assisted sputum suction. tracheostomy may improve outcomes by facilitating airway clearance, reducing ventilator-associated pneumonia (a common complication in IPA patients), and enabling earlier weaning from mechanical ventilation (Herritt et al., 2018; Chorath et al., 2021; Merola et al., 2024). However, this result may also be influenced by selection bias, as clinicians are more likely to perform this procedure on patients expected to survive longer.

Our findings differ from a recent study on COVID-19-associated invasive pulmonary aspergillosis in critically ill patients, which identified advanced age, mechanical ventilation, and high CRP levels as independent risk factors for mortality (Xiao et al., 2025). This discrepancy is likely attributable to differences in the study populations.

### 4.5 Strengths and limitations

Our study has several strengths that enhance its generalizability to similar tertiary care settings. As it was conducted in a large, mixed ICU cohort, the research captures real-world clinical diversity among critically ill patients beyond narrow, specific subpopulations. Our observed IPA incidence (7.1%) aligns with multicenter studies (5%–15%) in Europe and North America, and our diagnostic results match international meta-analysis. The application of standardized FUNDICU and EORTC/MSG criteria ensures our results are comparable across different geographical regions. Furthermore, our identification of IPA itself and delayed diagnosis as independent prognostic risk factors, provides actionable insights for ICU clinicians.

However, several limitations necessitate a nuanced interpretation of our findings. First, the single-center retrospective design may introduce potential selection and indication biases. In particular, the observed association between tracheostomy and improved survival likely reflects survivor bias rather than direct protective effect. Second, the scarcity of “proven” IPA cases—due to the inherent risks of invasive biopsy (Ledoux and Herbrecht, 2023)—along with the occasional lack of serial chest CT imaging, may have led to an underestimation of disease progression in our cohort.

## 5 Conclusion

IPA is a frequent and lethal complication in critically ill pneumonia patients, characterized by a shift from classical nerutropenia to broader iatrogenic host factors. BALF GM remains the diagnostic cornerstone, though a multimodal approach combing molecular tests is superior to traditional culture. Given the nonspecific and dynamic nature of CT findings, early and serial mycological screening is vital, our study demonstrates that while IPA independently increase mortality, early diagnosis and timely antifungal intervention significantly improve survival, highlighting the urgent need for high clinical suspicion and proactive testing in the ICU.

## Data Availability

The original contributions presented in the study are included in the article/supplementary material. Further inquiries can be directed to the corresponding author.
